# Ovarian Masses in Children and Adolescents: A Review of the Literature with Emphasis on the Diagnostic Approach

**DOI:** 10.3390/children10071114

**Published:** 2023-06-27

**Authors:** Effrosyni Birbas, Theofilos Kanavos, Fani Gkrozou, Chara Skentou, Angelos Daniilidis, Anastasia Vatopoulou

**Affiliations:** 1 Faculty of Medicine, School of Health Sciences, University of Ioannina, 45110 Ioannina, Greece; faybirbas@gmail.com; 2 Department of Obstetrics and Gynecology, Faculty of Medicine, School of Health Sciences, University of Ioannina, 45500 Ioannina, Greece; fani.gkrozou@uoi.gr (F.G.); haraskentou@uoi.gr (C.S.); anastasiavat@hotmail.com (A.V.); 3 1st Department of Obstetrics and Gynecology, Papageorgiou General Hospital, Faculty of Medicine, School of Health Sciences, Aristotle University of Thessaloniki, 56429 Thessaloniki, Greece; angedan@auth.gr

**Keywords:** ovarian masses, children, adolescents, tumor markers, imaging

## Abstract

Most abdominal masses in the pediatric population derive from the ovaries. Ovarian masses can occur in all ages, although their incidence, clinical presentation and histological distribution vary among different age groups. Children and adolescents may develop non-neoplastic ovarian lesions, such as functional cysts, endometrioma, torsion, abscess and lymphangioma as well as neoplasms, which are divided into germ cell, epithelial, sex-cord stromal and miscellaneous tumors. Germ cell tumors account for the majority of ovarian neoplasms in the pediatric population, while adults most frequently present with epithelial tumors. Mature teratoma is the most common ovarian neoplasm in children and adolescents, whereas dysgerminoma constitutes the most frequent ovarian malignancy. Clinical manifestations generally include abdominal pain, palpable mass, nausea/vomiting and endocrine alterations, such as menstrual abnormalities, precocious puberty and virilization. During the investigation of pediatric ovarian masses, the most important objective is to evaluate the likelihood of malignancy since the management of benign and malignant lesions is fundamentally different. The presence of solid components, large size and heterogenous appearance on transabdominal ultrasonography, magnetic resonance imaging and computed tomography indicate an increased risk of malignancy. Useful tumor markers that raise concern for ovarian cancer in children and adolescents include alpha-fetoprotein, lactate dehydrogenase, beta subunit of human chorionic gonadotropin, cancer antigen 125 and inhibin. However, their serum levels can neither confirm nor exclude malignancy. Management of pediatric ovarian masses needs to be curative and, when feasible, function-preserving and minimally invasive. Children and adolescents with an ovarian mass should be treated in specialized centers to avoid unnecessary oophorectomies and ensure the best possible outcome.

## 1. Introduction

Abdominal masses in the pediatric population most commonly derive from the ovaries [1]. Ovarian masses, including both non-neoplastic lesions and neoplastic tumors, can occur in all age groups. The incidence, clinical presentation and histological distribution of such lesions in children and adolescents are distinct from those in adults and require a particularized therapeutic approach [2]. Masses of the ovary range from simple functional cysts to malignant neoplasms. Ovarian malignancy is reported in 3–8% of children and adolescents with an adnexal mass and accounts for 1–2% of all childhood cancers [3,4]. During the evaluation of an ovarian mass, the most important objective is to assess the likelihood of malignancy since the management of benign and malignant lesions is essentially different. Therefore, the precise characterization of ovarian masses in children and adolescents is vital for appropriate treatment [3]. Several biomarkers, including imaging characteristics and serum tumor markers, can assist the diagnostic process and have been proposed as indicators of either high or low risk of malignancy. The differential diagnosis should be based on clinical manifestations, levels of serum tumor markers and imaging features.

Standard imaging modalities for the evaluation of a pediatric mass of the ovary include ultrasonography (US), magnetic resonance imaging (MRI) and computed tomography (CT). US is typically the initial modality of choice for assessing an adnexal mass since it is readily available and easy to use, does not expose young patients to ionizing radiation and does not require sedation. The transabdominal approach is generally used in the pediatric population, while endovaginal US may be used in sexually active adolescents [4,5]. However, the characterization of ovarian masses by US alone can be difficult in some cases and may require the use of additional modalities. MRI does not involve the use of ionizing radiation, provides excellent soft tissue contrast and offers the possibility for global abdominopelvic evaluation [3,4,6]. Therefore, it is ideal for the accurate characterization, localization and staging of pediatric ovarian masses. However, MRI is time-consuming, expensive, has limited availability and may require sedation in younger children [4,6,7]. Consequently, its use in the acute setting is limited [6]. CT, on the other hand, can easily be performed in emergent situations and, despite the radiation use, remains a useful diagnostic modality for the staging of neoplasms and surgical planning due to the aforementioned disadvantages of MRI [4,6]. Certain imaging characteristics of US, MRI and CT can be helpful in distinguishing benign from malignant ovarian masses (Table 1) [4,8,9,10,11,12,13,14].

Serum tumor markers are substances found in blood that may be elevated by the presence of a malignancy due to production either by the neoplasm itself or by normal cells in response to the neoplastic cells. Useful markers for the investigation of ovarian neoplasms in children and adolescents include alpha-fetoprotein (AFP), lactate dehydrogenase (LDH), beta subunit of human chorionic gonadotropin (β-hCG), cancer antigen 125 (CA-125) and inhibin (Table 2) [4,8,10,12,13,15,16,17,18,19]. Increased levels raise concern for cancer. However, elevated serum tumor markers can also be observed in benign lesions, and not all patients with a malignant neoplasm exhibit increased levels. Therefore, the measurement of tumor markers can neither confirm nor exclude malignancy with certainty. The diagnostic value of tumor markers is higher when the results are combined with clinical and radiological information. In any case, the definite diagnosis of an ovarian mass is established only after its surgical removal and histopathological examination [2]. Monitoring of serum tumor markers is helpful for postoperative follow-up as it may provide evidence of disease relapse [15].

Some types of pediatric ovarian neoplasms have been associated with specific cancer predisposition syndromes (Table 3) [20,21]. An ovarian neoplasm detected during childhood may be the first manifestation of a cancer predisposition syndrome, the diagnosis of which is of great importance since it provides the possibility of genetic counseling and surveillance of not only the patient but also her relatives. Hence, clinicians should always suspect the likelihood of a cancer predisposition syndrome when encountering ovarian neoplasms of specific histological types in children and adolescents to improve the prognosis of their patients and benefit their families [20].

Pediatric patients with an ovarian mass have a long-life expectancy after treatment, while the preservation of gonadal function is of paramount importance not only for fertility maintenance but also for the natural progression of puberty. In addition, abdominal scars can cause psychological issues and decreased self-confidence in young girls [2]. Hence, the management of ovarian masses in children and adolescents should be curative and, when feasible, function-preserving and minimally invasive, which can be achieved with laparoscopic ovary-sparing surgery [2,4,10]. The assessment of preoperative frailty and evaluation of possible predictors of pain development after surgery in these patients are fundamental for the prediction of and reduction in postoperative complications [22,23]. Imaging and serum tumor markers help in differentiating benign from malignant lesions and, thus, in determining the optimal treatment approach [8]. A multidisciplinary therapeutic team is necessary for the physical and psychological support of this vulnerable population of patients [13]. Although ovary-sparing operation is recommended for the management of benign ovarian masses in children and adolescents, 46–54% of such lesions are treated with oophorectomies. Patients treated by pediatric and adolescent gynecologists are less likely to undergo unneeded oophorectomies [24,25]. Consequently, pediatric patients with ovarian masses should be treated in specialized centers to ensure the best possible outcome.

Underneath in this review, we present the epidemiological features, clinical findings, diagnostic approach, management and prognosis of ovarian masses in children and adolescents with focus on the role of biomarkers, namely serum tumor markers and imaging characteristics.

## 2. Non-Neoplastic Lesions

Non-neoplastic masses of the ovary include benign tumor-like lesions that are not composed of neoplastic cells, such as functional cysts, endometrioma, torsion, abscess and lymphangioma.

### 2.1. Functional Cysts

Functional cysts, also known as physiological cysts, occur during the normal menstrual cycle and include follicular and corpus luteum cysts. Follicular cysts form when follicles fail to rupture during ovulation. Such cysts may develop due to a lack of the physiological release of the ovum as a result of excessive follicle-stimulating hormone (FSH) stimulation or absence of midcycle surge of luteinizing hormone (LH). They appear smooth, thin-walled and unilocular with a diameter larger than 3 cm. Corpus luteum cysts, on the other hand, arise when the dissolution of the corpus luteum does not occur. Under physiological conditions and unless pregnancy occurs, the functional life span of the corpus luteum is 14 days, after which it spontaneously involutes to form the corpus albicans, whereas, in cases of fertilization, the corpus luteum undergoes dissolution at 14 weeks of gestation. Corpus luteum cysts appear simple or complex, thick-walled, may contain internal debris and usually grow to 3 cm. Functional cysts are typically asymptomatic and spontaneously resolve without intervention [26].

Functional cysts are frequent among all pediatric age groups because of different hormonal insults. Causative mechanisms include the presence of maternal and placental hormones in infancy, the release of gonadotropins by the developing pituitary gland in the prepubertal period and dysfunctional ovulation in adolescence. The challenge in the management of girls with ovarian cysts lies in identifying a small percentage of neoplastic lesions among all these functional cysts [14].

**Figure 1:**
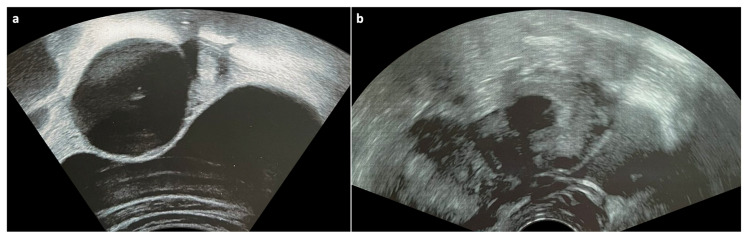
Ultrasonograms of two adolescents demonstrating (**a**) a thin-walled follicular cyst with posterior acoustic enhancement and (**b**) a thick-walled corpus luteum cyst.

As far as the sonographic appearance of functional cysts is concerned, follicular cysts are usually thin-walled with anechoic contents and rarely exceed 8–10 cm in diameter. Hyperechoic enhancement of the posterior wall is observed due to the reflection of the ultrasound beam off this wall after travelling through the anechoic window created by the clear contents of the cyst. Corpus luteum cysts appear thick-walled and hyperechoic (Figure 1). They typically exhibit peripheral circumferential blood flow, which is known as the “ring of fire sign”. The cyst contents typically exhibit a spider-web-like appearance due to limited internal hemorrhage, although they may also show different features, such as blood clots that resemble solid components. These clots have no blood flow in Doppler examination and present a typical jelly-like “wobbling” movement within the cyst when the ultrasound transducer is used to gently prod the ovary [27].

### 2.2. Endometrioma

**Figure 2:**
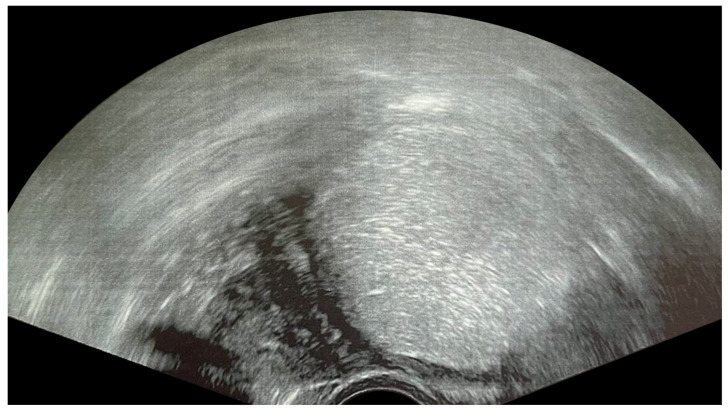
Ultrasonogram of a 17-year-old patient showing a typical unilocular endometrioma with ground-glass echogenicity of the cyst fluid.

Endometriomas are cystic lesions that arise from the disease process of endometriosis, which is defined as the presence of endometrial glands and stroma outside the uterus [28]. They contain a thick, dark brown, endometrial fluid that results from the accumulation of degenerated menstrual blood products and are, therefore, often referred to as chocolate cysts [29]. Endometriomas most commonly develop in the ovaries and can affect almost all ages, although they are typically diagnosed during the reproductive years [30,31]. Clinical manifestations include pelvic pain or tenderness, dysmenorrhea, dyspareunia, painful urination, painful defecation, urinary frequency, nausea/vomiting, back pain, palpable mass during bimanual examination in large enough lesions and acute abdomen in case of rupture [31]. Patients with any of these findings usually undergo an ultrasound examination, which, in case of ovarian endometrioma, classically reveals a simple, unilocular, avascular cyst with ground-glass appearance, namely low-level homogenous echoes that result from old hemorrhagic debris (Figure 2) [32]. On MRI, endometriomas typically appear T1 hyperintense without loss of signal after fat saturation and T2 hypointense. The low signal intensity on T2-weighted images, which is caused by the high concentration of protein and iron that result from cyclical hemorrhage, is referred to as the “shading sign” and, combined with high T1 signal intensity, is highly suggestive of endometrioma [33]. Treatment options for ovarian endometrioma include hormonal medications and surgery [31].

### 2.3. Ovarian Torsion

Ovarian torsion refers to the rotation of the ovary around the supporting ligaments, which can lead to obstructed lymphatic, then venous and finally arterial flow and infarction [6]. Adnexal torsion is a term used to describe the twisting of either the ovary, the fallopian tube or both [34]. Most of the times, both the ovary and a portion of the ipsilateral fallopian tube are affected, which is known as tubo-ovarian torsion, while isolated ovarian or isolated tubal torsion is rare [3,7,34,35]. Fifteen percent of cases of ovarian torsion occur in the pediatric population [7]. Although it can be observed in pediatric patients of any age, a bimodal age distribution has been described, with 16% of cases occurring in infants and 52% in the perimenarchal period [14,36].

Most pediatric patients with ovarian torsion have an underlying pathological condition in the involved adnexa that is present in 51–84% of cases. The underlying adnexal lesions are typically benign, with mature teratomas and follicular cysts most frequently detected, while malignant lesions associated with torsion are extremely rare in the pediatric population, possibly due to the presence of adhesions to the surrounding structures [6,7,36,37]. In a population of 86 girls aged up to 16 years studied by User et al. [14], torsion was more common among masses smaller than 6 cm and of cystic nature. These observations might be explained by the shallow pelvis of young girls, which hinders the mobilization of large masses and the greater gravitational force of solid portions found in neoplasms [14]. Torsion of normal adnexa without any underlying condition occurs more frequently in pediatric patients compared to adults [6]. A postulated mechanism for this condition is the increased mobility of adnexa due to the greater length and laxity of ligaments in some girls [6,7]. Ovarian torsion is rare after pelvic inflammatory disease (PID) and endometriosis probably because of the presence of adhesions that restrict ovarian mobility [34].

Clinical manifestations of ovarian torsion are often non-specific, mimicking other clinical entities of the genitourinary and gastrointestinal system. Most patients present with acute, unilateral, severe, lower abdominal pain, palpable mass, nausea/vomiting, fever and dysuria [6,34,38]. As mentioned above, 16% of pediatric ovarian torsion cases occur in infants. Ovarian abnormalities on prenatal imaging, abdominal mass and feeding intolerance have been described as the characteristic presenting clinical features in this age group [36].

US is the modality of choice for evaluating suspected ovarian torsion [3]. The most common sonographic finding of ovarian torsion in the pediatric population is the unilateral ovarian enlargement considered to be secondary to stromal edema and hemorrhage [6,38]. Sintim-Damoa et al. [6] suggest that torsion should be suspected when the ovarian volume is at least 3 times greater than expected according to the girl’s age. When considering whether an ovary is enlarged or not, comparing the size of the affected ovary with the size of the contralateral ovary is of great importance [6,7]. Other sonographic findings include peripherally displaced or even not visible follicles, medialization of the ovary, deviation of the uterus off the midline toward the twisted ovary, free pelvic fluid, a “whirlpool sign” of swirling vessels, coexistence of an adnexal mass and absent flow on Doppler examination [6,7]. However, the absence of Doppler flow cannot establish the absolute diagnosis because it can also be observed in normal, non-torsed ovaries in the pediatric population. In addition, present arterial flow cannot exclude the likelihood of ovarian torsion due to the dual ovarian blood supply from both the ovarian and uterine arteries and because arterial occlusion occurs after lymphatic and venous obstruction [6,7,38].

CT and MRI can be used as secondary imaging modalities for the evaluation of ovarian torsion. Both methods may show an enlarged torsed ovary with cystic appearance, possibly dislocated to the midline, while the uterus may be deviated toward the twisted ovary [3,6,7]. The MRI protocol should include fat suppression techniques to distinguish fat from hemorrhage and contrast-enhanced fat-suppressed T1-weighted images to help depict low enhancement of the ovary, which suggests infarction or necrosis [6,7].

In children and adolescents, ovarian torsion typically occurs acutely and constitutes a surgical emergency since it may result in impairment or loss of ovarian function due to persistent ischemia in cases of delayed diagnosis and intervention. Hence, it should be promptly detected and is treated with emergent, laparoscopic, surgical detorsion and visual inspection for viability in an attempt to salvage ovarian tissue [6].

### 2.4. Tubo-Ovarian Abscess

Tubo-ovarian abscess (TOA) is a complex, infectious, adnexal mass that results from PID and consequently affects sexually active patients usually aged 15–25 years [39]. It can manifest with abdominal pain, pelvic palpable mass, fever, nausea/vomiting, vaginal discharge or abnormal bleeding and cervical motion tenderness during bimanual examination. Further evaluation may show leukocytosis and bacterial growth in cervical, urine and blood cultures [40]. On imaging, TOA appears as a complex fluid-filled lesion with thick walls and septa [3]. US reveals an adnexal mass with mixed or ground-glass echogenicity [27]. On MRI, the fluid of TOA exhibits variable signal intensity on T1-weighted images, reflecting the degree of proteinaceous and hemorrhagic contents and often appears hyperintense on T2-weighted images. The walls and septa typically show low T1 and T2 signal intensity and enhance avidly [3]. T1-weighted imaging may demonstrate an internal hyperintense and enhancing rim, which is supposed to represent granulation tissue combined with hemorrhage [41]. Restricted diffusion is observed on diffusion-weighted imaging (DWI) [3]. Since TOA occurs in the context of PID, imaging can also depict adnexal edema and peritoneal fibrosis and adhesions, which appear as mesh-like linear strands with low T1 signal intensity and enhancement [41]. The combination of the aforementioned clinical and radiological characteristics render the diagnosis of TOA relatively straightforward. TOA is typically treated with antibiotics, although surgery may be unavoidable in some cases [40].

### 2.5. Lymphangioma

Lymphangiomas are rare benign malformations of the lymphatic system [42]. Their localization in the ovary is uncommon [43]. In fact, only 24 cases of ovarian lymphangioma have been reported in the literature during the last 70 years, the majority of which concerned adult patients [43,44,45,46,47,48,49,50,51,52,53,54,55,56,57,58,59,60,61,62,63,64,65,66]. Pani et al. [43] reported a case of a 16-year-old girl that presented with a longstanding, painless, abdominal distension and a non-tender, mobile, palpable mass of the entire abdomen. Tumor markers were negative. CT and MRI depicted a large, multiseptated, fluid-filled, cystic mass with well-defined and non-enhanced walls that measured 40 cm in the longitudinal axis and did not infiltrate into the surrounding tissues. The lesion was surgically removed, and the final diagnosis after histopathological examination was lymphangioma arising from the left ovary. The postoperative course of the patient was uneventful. This case highlights that lymphangiomas can occur in the pediatric population and should be included in the differential diagnosis of ovarian masses in this age group.

## 3. Neoplastic Tumors

Ovarian neoplasms are rare in the age group of our interest, with a reported annual incidence of 2.6 cases per 100,000 children and adolescents, and 90% of these tumors are proven benign [8]. Ovarian neoplasms are divided into germ cell, epithelial, sex-cord stromal and miscellaneous tumors, which account for 60–80%, 15–20%, 10–20% and less than 5% of cases in children and adolescents, respectively [4].

### 3.1. Germ Cell Tumors

Germ cell tumors, which originate from pluripotent germ cells, are the most common ovarian neoplasms in the pediatric population, accounting for approximately 60–80% of cases, with mature teratoma representing the predominant histological type, in contrast to adults, who most frequently present with epithelial tumors [16,18,19]. The majority of cases are benign [16]. Germ cell tumors are subdivided into mature teratoma, immature teratoma, dysgerminoma, yolk sac tumor, embryonal carcinoma, non-gestational choriocarcinoma, mixed germ cell tumor, gonadoblastoma and others [67].

#### 3.1.1. Mature Teratoma

Mature teratoma, also known as mature cystic teratoma or a dermoid cyst, is defined as a tumor containing exclusively mature tissues derived from two or three germ layers, namely ectoderm, mesoderm and endoderm [67]. It is among the most frequent ovarian neoplasms and exhibits a higher prevalence in the first two decades of life [68]. In fact, it is the most common ovarian neoplasm in children and adolescents, comprising more than 50% of cases in females younger than 20 years [19].

Although mature teratomas are mostly asymptomatic and can be diagnosed incidentally, patients may present with abdominal pain, palpable mass, sensation of abdominal fullness, constipation, nausea/vomiting and anorexia [2,4,69,70]. Complications of mature teratoma include torsion, rupture and infection and less commonly autoimmune hemolytic anemia, immune-mediated limbic encephalitis and malignant transformation [69,71,72,73,74,75,76,77,78,79].

Upon gross inspection, most mature teratomas are cystic masses surrounded by a capsule of variable thickness and typically filled with sebaceous liquid, while solid mature teratomas are rare [3,4,67,80]. They often present a focal protuberance projecting into the cyst, known as the Rokitansky nodule or dermoid plug, which may contain hair, bone, dental, muscle, calcific and cartilage components [3,4,80]. Comerci et al. [72] reported a mean tumor size of 6.4 cm in a population of 517 patients with mature teratoma. In pediatric patients, they are mostly unilocular and bilateral lesions are found in up to 10% of cases [4].

**Figure 3:**
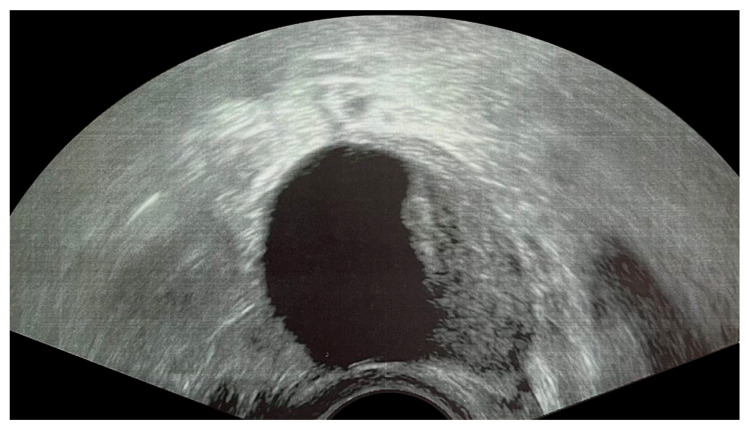
Ultrasonogram of an adolescent patient demonstrating a mature teratoma with a Rokitansky nodule that arises from the cyst wall and projects into the lumen.

US has become a highly effective modality for the diagnosis of mature teratoma. The most common sonographic appearance of mature teratoma is a cystic mass with a hyperechoic Rokitansky nodule that arises from the cyst wall and projects into the cyst lumen, often causing posterior acoustic shadowing (Figure 3) [4,16]. A Rokitansky nodule with posterior acoustic shadowing might be located in the superficial part of the lesion, hindering the sonographic visualization of the deeper structures of the mass. This sonographic appearance is referred to as the “tip of the iceberg sign” [4,16,81]. Other US findings include fat–fluid levels, also known as fluid–fluid levels, probably resulting from layered fatty sebum on top of serous fluid; the “dermoid mesh sign” referring to multiple hyperechoic lines and dots from hair within the cyst; echogenic mass representing sebaceous material and floating echogenic debris [4,16,82,83]. The sonographic appearance of mature teratoma may resemble bowel gas or fecal material in the colon, hemorrhagic ovarian cysts, endometrioma and perforated appendicitis and vice versa, occasionally leading to misdiagnosis [16,84].

**Figure 4:**
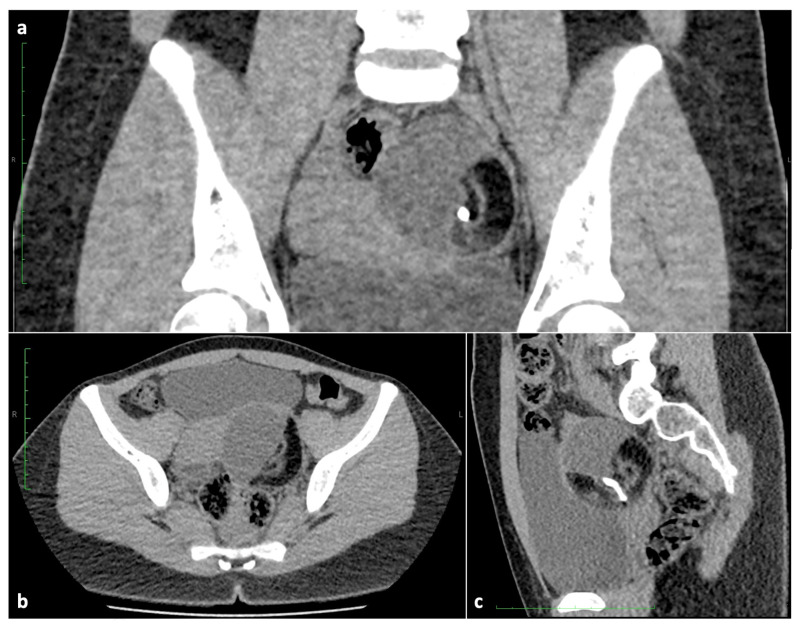
Computed tomography: (**a**) coronal, (**b**) axial and (**c**) sagittal scans of a 17-year-old patient showing a mature teratoma that contains fatty components, a Rokitansky nodule and a calcification.

Although US sets the diagnosis in most cases, CT or MRI should be considered in equivocal cases because both of them are more sensitive for fat detection [4]. CT can reveal cystic lesions with fat attenuation, calcification of the cyst wall, Rokitansky nodules, tufts of hair and fat–fluid levels, leading to a definitive diagnosis in 98% of cases (Figure 4) [10,85]. The visualization of multiple mobile globules composed of variable proportions of sebaceous material, fat, keratin, hair, fibrin and hemosiderin within an ovarian cystic lesion is referred to as the “floating balls sign” and can be recognized in both cross-sectional imaging and US. The term “Poké Ball sign” is used to describe a single large ball floating at the fat–fluid interface [84,86]. Although the “floating balls sign” is considered pathognomonic of mature teratoma, it is rarely observed, especially in the pediatric population, since it is identified in only 3.3% of girls [87].

**Figure 5:**
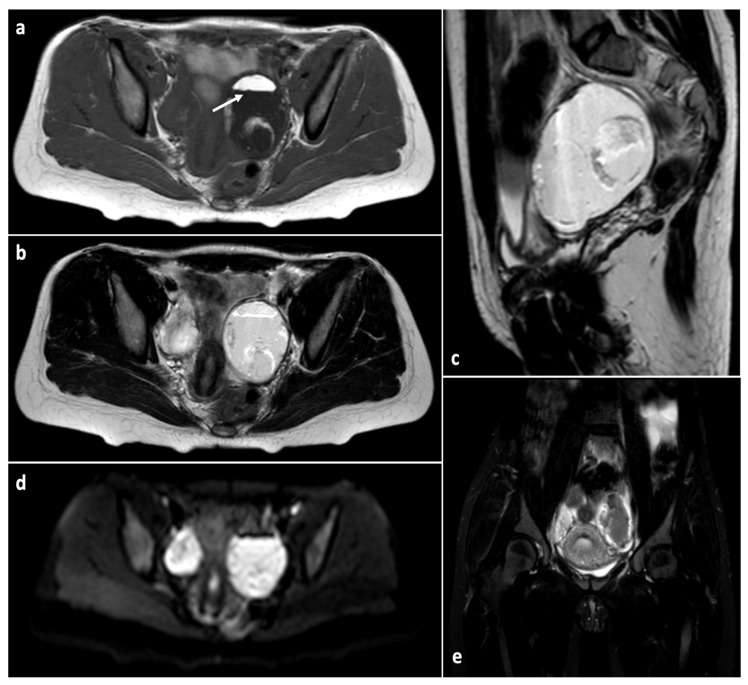
Magnetic resonance imaging sequences consistent with mature teratoma of the left ovary in an adolescent patient. (**a**) Axial T1-weighted image demonstrating the fat–fluid level (arrow) with hyperintense fat/sebaceous material on the non-dependent component of the mass; (**b**,**c**) axial and sagittal T2-weighted images, respectively, showing high signal of the cyst contents; (**d**) axial diffusion-weighted image revealing high signal of the dependent portion of the lesion; (**e**) coronal T2-weighted fat-suppressed image exhibiting signal drop within the mass.

On MRI, both fat in adipose tissue and lipid component in sebum present high T1 and intermediate to high T2 signal intensity. The presence of macroscopic fat can be confirmed by suppression of its signal on frequency-selective fat-saturated T1-weighted images, while signal drop on out-phase images can help to identify microscopic fat [3]. These fat suppression techniques can distinguish fat from other sources that shorten the T1 relaxation time, such as hemorrhage [3,16]. Deeply hypointense areas correspond to large areas of calcification or teeth. Fat–fluid levels can also be depicted via MRI [84]. The keratinoid substance is hypointense on T1-weighted images and hyperintense on T2-weighted images, resembling serous fluid. However, these two substances can be differentiated from each other via DWI since keratin content restricts the Brownian movement of water molecules, resulting in a high signal intensity on DWI and a low apparent diffusion coefficient (ADC) value (Figure 5). Hence, DWI is useful for the correct diagnosis of mature teratomas, especially in cases where fat is not obvious [84,88]. Solid components in the Rokitansky nodule can enhance without necessarily indicating malignant transformation [3].

Mature teratomas are benign neoplasms but may undergo malignant transformation in 0.17–2% of cases, typically in postmenopausal women and in lesions larger than 10 cm [69,79]. However, the malignant transformation is rare in the pediatric population [3]. Surgical treatment should be performed when the lesion becomes symptomatic or considered when it exceeds the diameter of 5 cm. The optimal surgical approach for pediatric patients combines laparoscopy and cystectomy maintaining as much ovarian tissue as possible [69].

Struma ovarii is a particular subtype of mature teratoma characterized by thyroid tissue as its predominant or sole component [67]. This ovarian neoplasm is primarily found in women aged 40–60 years and is extremely rare in children and adolescents. However, some cases concerning pediatric patients have been reported in the literature [89,90]. Most cases are asymptomatic, but some patients may present with abdominal pain, pelvic mass, ascites with or without pleural effusion and thyreotoxicosis [67,89,90,91]. Macroscopically, struma ovarii is usually unilateral and measures less than 10 cm. However, Ezon et al. [90] reported a case of a 16-year-old girl with a struma ovarii measuring 30 cm in its largest dimension. Most tumors are solid or cystic-solid, but some are mainly or entirely cystic [67,91]. Imaging features of this neoplasm are non-specific [92]. A “struma pearl” is a characteristic sonographic sign of struma ovarii referring to roundish hyperechoic areas of solid tissue with smooth surface and increased vascularization on Doppler examination that correspond to thyroid tissue [27,91]. Struma ovarii is typically a benign neoplasm of mostly solid nature; however, its clinical presentation is unspecific and can often mimic a malignancy [67,89]. When a solid mass of the ovary is encountered in a young girl, malignant neoplasms are suspected, but the likelihood of struma ovarii should not be ignored [89].

#### 3.1.2. Immature Teratoma

Immature teratoma also derives from more than one germ cell layer like mature teratoma but is composed of immature and variable amounts of mature tissues [3,67]. The relative quantity of immature elements determines the histological grade of the neoplasm [3]. It typically affects a younger age group, and the younger the patient, the higher the probability of a teratoma to be of the immature type [93]. This neoplasm usually presents within the first three decades of life and accounts for 10–20% of all ovarian malignancies in patients less than 20 years old [4,67]. In a population of 45 girls aged up to 15 years studied by Taskinen et al. [18], immature teratoma was second in frequency of ovarian neoplasms after mature teratoma and the most common ovarian malignancy. Pashankar et al. [94] calculated a mean age at presentation of 10 years within a pediatric population of 98 girls. Patients most often present with a palpable mass due to the large size of the tumor [16]. Immature teratomas present malignant behavior, grow rapidly, spread through the peritoneal cavity by implantation and metastasize primarily via lymphatics [80].

Regarding the radiological appearance of immature teratoma, it is usually unilateral, large, complex, heterogenous and mainly solid with cystic areas [4,95]. Tumor size is helpful in distinguishing immature from mature teratoma since their mean diameters are 16 cm and 6.5 cm, respectively [16]. Careful attention to the contralateral ovary should be paid as 10% of immature teratomas coexist with mature teratoma in the contralateral ovary [96]. In addition, ipsilateral mature teratomas are observed in 26% of immature teratoma cases [97]. Unlike mature teratomas, immature teratomas appear predominately solid with several microcystic components; contain scattered foci of macroscopic fat, the presence of which can be confirmed with fat suppression techniques, along with calcifications of small size and irregular shape; and occasionally present hemorrhage [95,97,98]. The microcystic structures are typically lined with respiratory epithelium and, hence, are filled with simple serous fluid with the corresponding MRI signal rather than sebaceous material, which is observed in mature teratomas. The amount of solid tissue demonstrated by imaging does not correlate with the histological grade of the tumor [95]. In cases of gliomatosis peritonei, which refers to the implantation of mature glial cells on the peritoneal surfaces usually in the context of immature teratoma, MRI can depict T2 hyperintense foci within the peritoneal cavity along with foci of nodular enhancement and ascites [16].

With regard to the role of tumor markers in the diagnostic procedure, immature teratoma has been associated with elevated AFP serum levels, which have been reported in 33–65% of patients [4,10,13,16]. Pashankar et al. [94] studied 89 pediatric cases of immature teratoma with available data on AFP levels and found them to be increased in 49% of patients. Nevertheless, increased serum levels of AFP in patients with apparent immature teratoma should prompt additional investigation and search with a focus on yolk sac tumor, the presence of which would change the diagnosis to mixed germ cell tumor [67].

Immature teratoma exhibits a more aggressive behavior and a poorer prognosis compared to mature teratoma, while disease stage and grade are considered significant prognostic factors [4,94]. The majority of children and adolescents with immature teratoma present with stage I or II disease, which is treated with complete surgical resection and exhibits a favorable prognosis [16]. Pashankar et al. [94] reported the clinical characteristics, treatment and outcomes of 98 pediatric patients with immature teratoma. Stage I disease was observed in 60% of cases. Ninety-two percent of patients were treated with surgery alone, whereas eight percent of them were treated with surgery plus postoperative chemotherapy. The five-year event-free survival (EFS) and overall survival (OS) rates were 91% and 99%, respectively. Relapse was observed in 9% of the patients, and the administration of postoperative chemotherapy was not found to decrease the risk of relapse.

#### 3.1.3. Dysgerminoma

Dysgerminoma, the ovarian analog of testicular seminoma, is a primitive germ cell tumor comprising cells showing no specific differentiation [3,4,67]. It is the most common ovarian malignant neoplasm in childhood and adolescence [3,4,8,9,16]. In a study of 94 female patients aged 10–20 years conducted by Gupta et al. [17], dysgerminoma was found to constitute 44% of all malignant ovarian tumors. The majority of cases occur in the second and third decades of life, with an average diagnosis age of 20 years, although 10% of cases are diagnosed during the first decade [4,16,99]. Dysgerminomas are often found in association with gonadal dysgenesis and abnormal ovaries that contain gonadoblastoma [4]. In fact, dysgerminoma is the most common ovarian malignancy in patients with gonadal dysgenesis [20]. An association between dysgerminoma and chromosomal abnormalities, such as Turner syndrome, has also been observed [4].

Most dysgerminomas are asymptomatic and quite large at diagnosis [100]. Clinical manifestations include abdominal pain, abdominal distention, palpable mass, decreased appetite, nausea/vomiting and ovarian torsion [101].

LDH and β-hCG are recognized as important tumor markers related to dysgerminoma [4,10,12,13,16,102]. LDH has been found to be elevated in up to 95% of patients and is useful for diagnosis and postoperative follow-up [4,103]. Serum β-hCG levels are rarely elevated in approximately 5% of cases, particularly in the presence of syncytiotrophoblastic giant cells [4,10,104].

Upon gross pathological inspection, dysgerminoma is commonly well-encapsulated, lobulated and solid with white to light tan color. Necrosis, cystic changes, calcifications and hemorrhagic areas can be observed within the tumor. At histological examination, sheets and nests of monotonous neoplastic cells are separated by fibrous or fibrovascular septa [4,67].

The characteristic imaging presentation of dysgerminoma is a heterogenous, multilobulated, solid lesion that contains prominent fibrovascular septa [105]. The mass is hypointense to muscle on T1-weighted images and isointense to slightly hyperintense on T2-weighted images. The septa that course through the mass appear hypoechoic on ultrasound examination and hypointense on T2-weighted images because of their fibrous content and show intense enhancement with contrast administration [105,106]. In addition, blood flow within the septa can be depicted with Doppler examination. Other imaging findings associated with dysgerminoma include necrotic and hemorrhagic foci and speckled calcifications [106]. Dysgerminomas are bilateral in 10–15% of cases and can spread to the retroperitoneal lymph nodes. Consequently, the contralateral ovary and the possible presence of nodal metastases should be evaluated at diagnosis and follow-up [4].

With regard to the treatment of dysgerminoma, effective therapeutic options include conservative surgery, chemotherapy and irradiation [100,107]. Most patients present with early-stage disease, and, hence, surgery alone can be curative. The prognosis of dysgerminoma is usually excellent [4]. De Bénazé et al. [107] reported the clinical outcomes of 48 pediatric patients aged up to 18 years with dysgerminoma. Treatment was based on primary unilateral oophorectomy followed by prophylactic lymph node irradiation or a wait-and-see strategy for localized completely resected tumors or platinum-based chemotherapy for advanced cases. All patients were alive with complete remission after a median follow-up of 14 years. The authors concluded that dysgerminoma presents an excellent prognosis with conservative surgery and chemotherapy even in patients with advanced disease, although a high rate of bilateral oophorectomies and an important impact on future fertility were observed due to the disease and/or its treatment.

#### 3.1.4. Yolk Sac Tumor

Yolk sac tumor, formerly known as endodermal sinus tumor, is a primitive germ cell tumor exhibiting multiple patterns reflecting endodermal extraembryonal differentiation or endodermal somatic tissues [67]. Cases most commonly occur in the second and third decade of life with an average diagnosis age of 19 years [4]. The occurrence of this specific neoplasm is generally uncommon [3]. For example, in the study of 94 female patients aged 10–20 years conducted by Gupta et al. [17], only two cases of yolk sac tumor were observed, which accounted for 7% of all ovarian malignancies. However, a unique histological distribution of ovarian neoplasms was reported by AlDakhil et al. [9], who studied 164 Saudi Arabian female patients aged 10–20 years and found that yolk sac tumor represented the most common ovarian malignancy, constituting 40% of all malignant cases.

Yolk sac tumors, along with mixed germ cell tumors with yolk sac components, produce high levels of AFP, which can be of great assistance for the initial diagnosis and postoperative follow-up of patients [4,10,13,15,18,102,108]. In the study of 45 girls by Taskinen et al. [18], all three patients diagnosed with yolk sac tumor exhibited elevated AFP. In addition, patients with yolk sac tumors or mixed germ cell tumors with yolk sac components presented the highest serum AFP levels.

The neoplasm is macroscopically described as a large, solid and cystic, unilateral lesion with friable, hemorrhagic and necrotic appearance [67].

Yolk sac tumors radiologically appear as heterogenous, large, mixed solid and cystic masses with intratumoral hemorrhage, necrosis and striking enhancement [109,110,111,112]. The internal hemorrhage and intense enhancement reflect the rich vascularity of the neoplasm [3]. A characteristic imaging finding of yolk sac tumors is the depiction of large dilated vessels or vascular aneurysms, which can be demonstrated as signal voids on spin echo MRI or directly following contrast medium administration [3,4]. Ascites may also be noticed. Although the aforementioned imaging findings are unspecific, the possibility of a yolk sac tumor should be considered in cases of young patients with increased serum AFP levels and a large, mainly solid ovarian tumor [4].

Yolk sac tumors are usually aggressive, exhibit malignant behavior, grow rapidly and present peritoneal, hematogenous and lymphatic metastases [3,4]. Most cases of this neoplasm can be treated with conservative surgery and multiagent chemotherapy, and the prognosis depends on the stage of the disease [4].

#### 3.1.5. Embryonal Carcinoma

Embryonal carcinoma is a primitive malignant germ cell tumor that may display somatic or extraembryonal differentiation [67]. It is rare and mainly affects children and adolescents with a median diagnosis age of 14 years. It mostly occurs as a component of a mixed germ cell tumor, whereas its pure form is extremely uncommon. Patients present with abdominal pain and palpable mass, while endocrine alterations, including isosexual precocious puberty and menstrual abnormalities related to the secretion of β-hCG, are observed in up to 60% of cases [113]. This neoplasm may produce AFP and/or β-hCG, which can assist diagnosis and treatment monitoring [3,10,13,102,113]. Upon macroscopic pathologic inspection, the neoplasm appears as a unilateral, large, primarily solid, variegated and extensively hemorrhagic and necrotic mass with cystic structures containing mucoid material [113]. Imaging presentation is unspecific and similar to that of other malignant germ cell tumors [114].

#### 3.1.6. Non-Gestational Choriocarcinoma

Non-gestational choriocarcinoma is defined as a malignant germ cell tumor that consists of the cytotrophoblast and syncytiotrophoblast and is not of gestational origin [67]. It is extremely rare and primarily affects children, adolescents and young adults [115,116]. Clinical manifestations include vaginal bleeding, abdominal pain, palpable pelvic mass and isosexual precocious puberty [115]. Serum β-hCG levels are typically elevated [3,4,10,13,15,102,115,117]. The major treatment strategy for non-gestational choriocarcinoma is surgery combined with chemotherapy. Early metastasis is commonly observed, and the disease prognosis is poor [115].

#### 3.1.7. Mixed Germ Cell Tumor

Mixed germ cell tumors are defined as neoplasms that consist of at least two malignant germ cell components. They predominately concern children and young women. Typical clinical manifestations include abdominal pain and mass, menstrual disorders and peripheral precocious puberty. Tumor markers, namely LDH, β-hCG and AFP, may be positive depending on the nature of the components present inside the tumor [67]. Imaging presentation reflects the histological components within the lesion. Mixed germ cell tumors typically appear as large, mainly solid masses with extensive areas of hemorrhage and necrosis [3].

#### 3.1.8. Gonadoblastoma

Gonadoblastoma is a rare neoplasm composed of germ cells mixed with incompletely differentiated sex cord cells and belongs to the category of germ cell-sex-cord stromal tumors of the ovary. It is restricted to patients with gonadal maldevelopment and affects 50–60% of patients with dysgenetic gonads [67]. Gonadoblastoma has been described in 10–35% of patients with Turner syndrome with Y chromosome material, namely 45,X/46,XY mosaicism. WT1-related disorders, which include Frasier, Denys–Drash and WAGR syndromes, lead to gonadal dysgenesis and have been associated with gonadoblastoma. Frasier and Denys–Drash syndromes both manifest with nephropathy, male pseudohermaphrodism and gonadal tumors, with gonadoblastoma occurring in approximately 37–60% and 4% of cases, respectively. WAGR syndrome is mainly characterized by Wilms tumor, aniridia, genitourinary anomalies and intellectual disability and has been described in children with gonadoblastoma to a lesser extent compared to Frasier and Denys–Drash syndromes [20].

Most cases of gonadoblastoma are diagnosed during the investigation for a suspected disorder of sex development, usually due to ambiguous external genitalia in infants [67]. Gonadoblastoma can be hormonally active, and, therefore, patients may present with precocious puberty or virilization due to estrogen or androgen production, respectively [118]. Clinical features of the aforementioned syndromes may also lead to the discovery of this tumor.

Imaging findings of gonadoblastomas are poorly established because these neoplasms can be too small to be detected by diagnostic modalities [119]. Most macroscopically obvious lesions appear solid with calcifications [10,119,120]. Bilaterality is observed in up to 50% of cases [4,10,121].

Gonadoblastoma is cured with surgical excision, and bilateral oophorectomy is recommended in the presence of abnormal contralateral ovary due to the high rates of bilateral neoplasms in these cases [119]. This neoplasm presents a favorable prognosis; however, some cases are associated with a coexisting malignant germ cell tumor, particularly dysgerminoma [4,118].

### 3.2. Epithelial Tumors

Epithelial tumors are rare in children and account for only 15–20% of all pediatric ovarian neoplasms, although they represent the most common ovarian tumors in adult women [4,16]. They occur almost exclusively after menarche as their development might be triggered by hormonal stimulation [122]. In a series of 19 patients aged less than 21 years with an epithelial ovarian tumor, Tsai et al. [122] found a median age at diagnosis of 19.7 years. These neoplasms are divided into serous, mucinous, endometrioid, clear cell, seromucinous and Brenner tumors, and each subtype can be classified as benign, borderline or malignant [67]. Borderline or low malignant potential tumors exhibit nuclear atypia and increased mitotic activity but lack stromal invasion [16]. Serous and mucinous neoplasms constitute the vast majority of epithelial tumors of the ovary in the pediatric population [122,123]. In children and adolescents, epithelial tumors are mostly benign and less frequently borderline, whereas malignant cases are scarce [123,124]. In a study of 69 patients aged up to 18 years with an epithelial tumor of the ovary, Hazard and Longacre [124] found that benign, borderline and malignant neoplasms accounted for 57.8%, 37.5% and 4.7% of cases, respectively. It is worth mentioning that borderline neoplasms are more common in children compared to adults [4]. Common presenting symptoms include dysmenorrhea and abdominal pain [122].

The value of tumor markers in the diagnostic process of pediatric epithelial ovarian tumors is limited. Elevated serum CA-125 levels are typically associated with epithelial malignancies of the ovary [4,10,13]. However, they exhibit a lower sensitivity and specificity for the detection of malignant epithelial ovarian tumors in premenarchal girls than in adults [10]. CA-125 levels should not be routinely measured in children and especially in premenarchal girls since epithelial tumors of the ovary are exceedingly uncommon before menarche [4].

Benign, borderline and malignant epithelial ovarian tumors exhibit different imaging features that may aid differential diagnosis. Cystadenomas, which are benign neoplasms, represent cystic lesions with thin walls, septa less than 3 mm in thickness when present and no solid components [125,126]. Serous cystadenomas typically appear as unilocular cystic masses that lack septa and may be radiologically indistinguishable from follicular cysts, although they occasionally contain one or two septa and a few locules [3,16]. Contents of uncomplicated cysts exhibit signal characteristics similar to those of simple fluid [3]. Mucinous cystadenomas, on the other hand, are usually multilocular [16,126]. Cyst contents present variable signal intensity on MRI and attenuation on CT depending on their mucinous composition, which results in the classic but not specific stained-glass appearance of the numerous locules on T2-weighted images [3,126,127].

Imaging findings suggestive of borderline or malignant neoplasms include larger size, complex morphology, thick and irregular walls, septa thicker than 5 mm and solid components with necrosis [4,126]. Papillary projections, which represent folds of the proliferating neoplastic epithelium growing over a central fibrovascular stromal core and need to be differentiated from mural solid tissue, are distinctive features of epithelial ovarian tumors and especially characteristic of borderline masses, although they can also be present in malignant and less commonly in benign lesions [16,126,128]. Papillary projections exhibit intermediate signal intensity along with avid post-contrast enhancement on T1-weighted images and a central hypointense core surrounded by a hyperintense periphery on T2-weighted images [128,129]. Carcinomas typically contain predominant solid portions. Supplemental findings of malignancy include lymphadenopathy, peritoneal implants, ascites and direct local invasion [3].

Benign epithelial ovarian tumors are treated with conservative surgery [4]. Borderline and malignant neoplasms are usually low-grade and early-stage in children and adolescents and, thus, possess a better prognosis compared to adults [4,122,124]. Conservative surgery is suggested for localized cases, whereas advanced tumors require more intense treatment [4].

### 3.3. Sex-Cord Stromal Tumors

Sex-cord stromal tumors arise from sex cord cells, namely granulosa and Sertoli cells, and stromal cells, namely fibroblasts, theca cells and Leydig cells, and represent 10–20% of all pediatric ovarian neoplasms [4,130]. They are often associated with more than one cell type and are subdivided into three subgroups: pure sex cord tumors, pure stromal tumors and mixed sex-cord stromal tumors [67]. These neoplasms can develop in females of all ages; however, the histological distribution is largely different among age groups. Juvenile granulosa cell tumor and Sertoli–Leydig cell tumor are the most common types in children and adolescents, whereas thecoma and fibroma are rare [131,132]. The opposite applies for adult women, among whom fibroma predominates [3,4]. Sex-cord stromal tumors are clinically significant due to their hormonal activity as juvenile granulosa cell tumors, and thecomas typically secrete estrogens, while Sertoli–Leydig cell tumors frequently produce androgens [4].

#### 3.3.1. Juvenile Granulosa Cell Tumor

Granulosa cell tumors are subdivided into adult and juvenile types based on their clinical and histopathological characteristics. Juvenile granulosa cell tumor is a malignant pure sex cord tumor consisting of primitive-appearing granulosa cells that grow in solid and follicular patterns [67]. It accounts for 5% of granulosa cell tumors and constitutes 70% of all sex-cord stromal tumors in patients less than 20 years old [133,134]. It mainly occurs in patients younger than 30 years with a mean age at diagnosis of 13 years [16,135]. Young et al. [135] studied 125 patients with juvenile granulosa cell tumor and reported that 44% of cases occurred in the first and 34% in the second decade of life. Several studies have provided evidence that juvenile granulosa cell tumor is associated with Ollier disease and Maffucci syndrome, which are subtypes of enchondromatosis [20,136,137,138,139,140,141,142,143,144]. These conditions are characterized by the presence of multiple, asymmetrically distributed enchondromas, with Maffucci syndrome also including soft tissue hemangiomas unlike Ollier disease [143,145].

Patients with juvenile granulosa cell tumor may present with manifestations related to a pelvic mass and hormonal disturbances. Acute complications, including torsion and rapture, are more frequently observed in children compared to adults [4]. Juvenile granulosa cell tumors typically secrete estrogens [3]. As a result, premenarchal patients usually present with clinical manifestations of isosexual peripheral precocious puberty, including breast enlargement, vaginal bleeding, axillary and pubic hair and somatic growth, while postmenarchal patients may complain of menstrual disorders, such as menorrhagia or amenorrhea [146]. Rare cases of virilization due to production of androgens by the neoplasm have also been reported [4,16,146].

Granulosa cells produce inhibin, and, thus, serum inhibin levels can be elevated in patients with juvenile granulosa cell tumor. Consequently, inhibin is considered a useful tumor marker in terms of diagnosis and follow-up [4,10,15,16].

Imaging features of this neoplasm are heterogenous and unspecific [3]. Juvenile granulosa cell tumors are typically large, and most of them contain both solid and cystic components, although they may be entirely solid or completely cystic [147,148,149]. Intratumoral hemorrhage, necrosis and fibrosis are commonly observed [3]. T2-weighted MRI sections may reveal a sponge-like appearance of the neoplasm formed by solid areas of intermediate signal intensity intermixed with multiple cystic spaces [148,149,150]. T1-weighted images, on the other hand, can often depict hyperintense hemorrhagic foci within the cysts [130,149,150,151]. Endometrial thickening and uterine enlargement can be demonstrated as a result of estrogen secretion [148,149].

Prognosis of juvenile granulosa cell tumor is favorable, and late recurrences are rare [134]. The majority of patients present with early-stage disease limited to the ovary and have an excellent prognosis with a survival rate of over 90% with surgery alone [152]. However, rare cases with advanced disease exhibit a poorer prognosis and may require chemotherapy [4].

#### 3.3.2. Sertoli–Leydig Cell Tumor

Sertoli–Leydig cell tumor is a rare mixed sex-cord stromal tumor that consists of varying proportions of Sertoli and Leydig cells. It is subdivided into well, moderately and poorly differentiated forms [67]. Moderately and poorly differentiated neoplasms are clinically malignant in approximately 10% and 60% of cases, respectively, and may contain retiform patterns as well as heterologous elements, such as mucinous enteric epithelium or hepatocytes [16,21,153,154]. Sertoli–Leydig cell tumors usually affect females younger than 30 years old with a median diagnosis age of 14 years [134]. Young and Scully [153] studied 207 patients and found that 6% of cases occurred in the first and 46% in the second decade of life. Sertoli–Leydig cell tumors of moderate and poor differentiation exhibit a high association with DICER1 syndrome, which is characterized by germline mutations of the DICER1 gene [16,20]. In a study of 34 patients with this neoplasm conducted by de Kock et al. [21], all moderately and poorly differentiated cases and none of the well differentiated cases were found to be DICER1-mutated. These findings provide evidence that DICER1 mutation may be a defining feature of moderately and poorly differentiated tumors, whereas well differentiated neoplasms seem to be DICER1-independent [21]. Although a definitive link has not been established, Sertoli–Leydig cell tumors have been described in children and young women with Peutz–Jegher syndrome, and further studies may confirm or disprove this association [20].

Clinical manifestations include abdominal palpable mass and pain as well as hormonal abnormalities [15,20]. Sertoli–Leydig cell tumor is the most common androgen-producing neoplasm of the ovary, and it frequently leads to features of virilization, such as hirsutism [130,153]. Estrogen production, which results in precocious puberty and menstrual disturbances, is rare [153].

With regard to the role of tumor markers in the investigation of Sertoli–Leydig cell tumors, elevated serum levels of AFP can rarely be observed as a result of the presence of heterologous hepatocyte elements [133,154].

On imaging, Sertoli–Leydig cell tumor typically appears as a predominately solid mass with numerous peripheral or intratumoral cysts or as a cystic mass with solid mural components [4,130,147,155]. The MRI signal intensity of solid portions varies depending on the amount of underlying fibrous tissue but is typically low on T1-weighted images and low to intermediate on T2-weighted images. The lesion presents intense enhancement after administration of contrast medium [130,155].

Prognosis of Sertoli–Leydig cell tumors is based on the stage and grade of the disease [156]. Most cases are diagnosed as early-stage and low-grade neoplasms with favorable prognosis; however, recurrences are common, and higher relapse rates are noted in poorly differentiated neoplasms [3,4,156]. In a series of 44 pediatric patients with a median age of 13.9 years, Schneider et al. [156] reported an overall survival of 87% after a median follow-up of 62 months. All patients underwent surgical resection, and some received cisplatin-based chemotherapy [156]. It is recommended to perform germline DICER1 mutation testing in all patients with Sertoli–Leydig cell tumor since a positive result is important for follow-up and genetic counseling [15,21].

### 3.4. Miscellaneous Tumors

Miscellaneous tumors of the ovary include the remaining neoplasms that do not belong to the above categories. Ovarian metastases in childhood are usually hematogenous in contrast to those found in adult patients [3]. McCarville et al. [157] studied 18 children with ovarian metastases and reported that half of the cases derived from colon adenocarcinoma and the remaining ones from alveolar rhabdomyosarcoma, Burkitt lymphoma, Wilms tumor, neuroblastoma and retinoblastoma.

Small cell carcinoma of the ovary, hypercalcemic type, is an undifferentiated malignant neoplasm consisting of small cells with or without a large cell portion that is unrelated to small cell neuroendocrine carcinoma [67]. It is uncommon and, according to Young et al. [158], approximately 75% of cases occur during the second and third decades of life with an average diagnosis age of 23.9 years. Paraneoplastic hypercalcemia is observed in about two thirds of patients [20,158]. This neoplasm is largely associated with germline or somatic mutations in the chromatin remodeling gene SMARCA4 [15,159,160]. Germline mutations of this gene predispose carriers to develop rhabdoid tumors too, which most frequently result from mutations in SMARCB1, another chromatin remodeling gene [161,162]. SMARCB1 and SMARCA4 germline mutations define rhabdoid tumor predisposition syndrome types 1 (RTPS1) and 2 (RTPS2), respectively. Small cell carcinoma of the ovary, hypercalcemic type, is morphologically similar to rhabdoid tumors, suggesting that it belongs to the rhabdoid tumor spectrum. Given the high prevalence of RTPS2 in patients with this rare neoplasm, it is recommended that all patients should be referred to a specialist to receive genetic counselling [20]. On imaging, the tumor appears as a solid enhancing lesion occasionally with cystic and hemorrhagic foci [16]. It is an extremely aggressive neoplasm with poor prognosis in spite of combined surgery and aggressive chemoradiotherapy [16,20,67].

## 4. Conclusions

Ovarian masses in children and adolescents range from simple functional cysts to malignant neoplasms. Their incidence, clinical presentation and histological distribution are distinct from those in adults and require a particularized therapeutic approach. Germ cell tumors represent the majority of ovarian neoplasms in children and adolescents, while adults most frequently develop epithelial tumors. The most common ovarian neoplasm in the pediatric population is mature teratoma, whereas dysgerminoma constitutes the most frequent ovarian malignancy. Imaging and serum tumor markers can assist the diagnostic process and help in differentiating benign from malignant lesions and, thus, in determining the optimal treatment options. Preserving gonadal tissue is of paramount importance in children and adolescents not only for fertility maintenance but also for the natural progression of puberty. Management of pediatric ovarian masses needs to be curative and, when feasible, function-sparing and minimally invasive. Children and adolescents with an ovarian mass should be treated by multidisciplinary teams in specialized centers, which are able to provide the optimal physical and psychological support, avoid unnecessary oophorectomies and ensure the best possible therapeutic result.

## Figures and Tables

**Table 1:** Imaging findings of ovarian masses that suggest either high or low risk of malignancy.

Characteristic	Malignant	Benign
Consistency	Solid or presence of solid components	Cystic
Size	Large (≥8–10 cm)	Small (<8–10 cm)
Composition	Heterogenous	Homogenous
Ovarian torsion	Less likely	More likely
Papillary projections	More likely	Less likely

**Table 2:** Serum tumor markers and associated pediatric ovarian neoplasms.

Tumor Marker	Ovarian Neoplasm
AFP ^1^	Immature teratoma Yolk sac tumor Embryonal carcinoma Sertoli–Leydig cell tumor (rare)
LDH ^2^	Dysgerminoma
β-hCG ^3^	Dysgerminoma (rare) Embryonal carcinoma Non-gestational choriocarcinoma
CA-125 ^4^	Malignant epithelial tumors
Inhibin	Juvenile granulosa cell tumor

^1^ alpha-fetoprotein, ^2^ lactate dehydrogenase, ^3^ beta subunit of human chorionic gonadotropin, ^4^ cancer antigen 125.

**Table 3:** Pediatric ovarian neoplasms and associated cancer predisposition syndromes.

Ovarian Neoplasm	Cancer Predisposition Syndrome
Gonadoblastoma	Frasier syndrome ^1^ Denys–Drash syndrome ^1^ WAGR syndrome ^1^ 45, X/46, XY mosaicism
Juvenile granulosa cell tumor	Ollier disease ^2^ Maffucci syndrome ^2^
Sertoli–Leydig cell tumor	DICER-1 syndrome
Small cell carcinoma of the ovary, hypercalcemic type	Rhabdoid tumor predisposition syndrome type 2

^1^ WT1-related disorder, ^2^ subtype of enchondromatosis.

## Data Availability

No new data were created or analyzed in this study. Data sharing is not applicable to this article.
